# Unraveling the Mystery of “The Specificity of Women’s Sexual Response and Its Relationship with Sexual Orientations”: The Social Construction of Sex and Sexual Identities

**DOI:** 10.1007/s10508-017-0957-x

**Published:** 2017-02-21

**Authors:** Jane M. Ussher

**Affiliations:** Centre for Health Research, School of Medicine, Western Sydney University, Sydney, NSW 2751 Australia

In a critical review and of research examining women’s sexual response across a range of sexual orientations, Chivers (2017) draws a number of conclusions. Heterosexual women, whom she describes as “androphilic,” respond in equal measure to sexual representations of both men and women, characterized as a “gender-nonspecific pattern of sexual response.” Lesbian women, described as “gynephilic,” respond primarily to images of women, a “gender-specific response.” The few studies of bisexual or “ambiphilic” women suggest greater response to images of women, but Chivers concludes that there are insufficient data for firm conclusions to be drawn. Both heterosexual and gay men report a gender-specific sexual response commensurate with their declared sexual orientation, the former responding to representations of women and the latter to men.

## Confined by a “Straightjacket of Biological Reductionism”: Limitations of a Positivist–Realist Epistemology

The research analyzed in Chivers’ review is positioned firmly within a positivist/realist epistemology, reflecting the penchant of sexologists to utilizing the “rigorous” experimental methods of the natural sciences, in order to maintain legitimacy and separate their analysis from politics and the “fuzzy humanities” (Tiefer, 1992). This includes experimental research on visual attention to sexual images, implicit and explicit cognitive processing, affective processing, genital sexual arousal responses, activation of the autonomic nervous system, and reward assessment. The few studies which include subjective evaluation are implicitly positioned as less reliable or valid, evidenced by Chivers’ comment “it is worth noting that these data were self-reported and observational, not experimental.” This methodology reflects the narrow conceptualization of sex enshrined within the Masters and Johnson Human Sex Response Model (HSRC) (Masters & Johnson, 1966). As Tiefer (2004) has argued, this results in a conceptualization of sexuality as “the performance of fragmented body parts” (p. 53), focusing on physiological response and the genitals, while denying social and relational context. The meaning of sexual response, sexual desire, and sexual “orientation” is not part of this reductionist equation, and sex is conceptualized outside of cultural discourse, as something that can be legitimately studied in a laboratory. In the adoption of what Tiefer (1991) has described as a “phallocentric straightjacket of biological reductionism” (p. 27), understanding of the complexity and meaning of women’s sexual response is thus sacrificed to the holy grail of scientific objectivity.

Chivers completes her review with an evaluation of ten “hypotheses” which could explain why “androphilic women continue to be a mystery,” because their professed “sexual orientation” is at odds with their sexual response. The assumption that one model or “hypothesis” could provide a complete explanation of the reported findings reflects the positivist focus on unilinear notions of cause and effect (Keat, 1979), negating the complexity and multiplicity of sexual response and sexual orientation (Bancroft & Graham, 2011; Weeks, 2003). The positivist emphasis on facts and negation of values also results in an absence of reflexivity (see Chamberlain, 2004; Finlay & Gough, 2003). There are no discussion of the subjectivity, gender, and sexual orientation of the individuals who conducted the research; no critical reflection on the artificiality of the research context, wherein sexual response is elicited and measured in an experimental setting; no discussion of the participants who take part in such research-primarily North American college students or small numbers of “adult volunteers.” Women who participate in sex research have been reported to have had more sexual trauma, to masturbate more frequently, and to have had greater exposure to pornography at an early age, as well as less sexual fear than found in the general female population (Wolchik, Braver, & Jensen, 1985). We may thus question the generalization of their experiences and responses to the population of women as a whole.

The HSRC implicitly reifies the phallocentric “coital imperative” that positions penis–vagina penetration as “real sex” (McPhillips, Braun, & Gavey, 2001), within “compulsory heterosexuality”—the taken-for-granted sexual identity position (Rich, 1980). (Hetero)sexual acts such as mutual masturbation, cunnilingus, or fellatio are often deemed “foreplay” and positioned as not “having sex” (Gavey, McPhillips, & Braun, 1999); however, they are an acknowledged part of the arousal stage of the HSRC and thus included in the experimental research Chivers’ reviews, expected to elicit “normal” (hetero)sexual response. Sexual acts between women are not deemed “normal” for avowedly heterosexual women, leading Chivers to conclude that “androphilic women continue to be a mystery” in sexually responding to such imagery. From a social constructionist perspective, there is no mystery.

## Explaining the “Mystery” of Heterosexual Women’s Gender-Nonspecific Sexual Response: A Social Constructionist Analysis

Within a social constructionist perspective, experiences of sexual response and orientation are understood as learned phenomena mediated by social, cultural, and intersubjective factors (Tolman, 2002; Ussher, 1997). The very notion of “sex” is socially constructed, with particular bodily acts and sexual identity positions positioned as legitimate or as deviant within specific social and historical contexts (Foucault, 1978; Plante, 2015). We therefore need to look at the cultural and relational context of the “androphilic” women taking part in experimental sex research, in particular their discursive constructions of sex and sexual identity, in order to understand Chivers’ findings of gender-non-specific sexual response.

In many non-Western cultures, participation in the experimental sex research that Chivers’ reviews would be considered culturally unacceptable, with women not expected to know or talk about sex, and certainly not to view or respond to visual images of naked or copulating couples, or to exhibit same-gender desire or response (Ussher et al., 2017). In contrast, in Western societies, the proliferation of “raunch culture” is associated with increased acknowledgment of women’s agentic sexuality (Bale, 2011), including the visibility of “girl-on-girl” sex, in television shows, movies, and “girls gone wild” videos (Levy, 2005; Thompson, 2006). There is evidence that an increasing number of Western women access pornography (Attwood, 2005; Rissel et al., 2017), including the eroticized images of lesbian sex that appear within heterosexual porn (Ussher, 1997). Prepubescent and pubescent girls commonly engage in same-gender sexual exploration (Lamb, 2004), adult women in “passionate friendships” (Glover, Galliher, & Crowell, 2015), and a high proportion of college age women in North America have experienced same-gender kissing or “making out” (Fahs, 2009; Meyer, 2005).

However, same-gender sexual activity is not necessarily evidence of exploration of lesbian or bisexual identity or, indeed, a reflection of women’s sexual desire (Alarie & Gaudet, 2013). College age and adult women report engaging in sexual activities with other women as a performance for men in order to gain attention, or in response to demands from a male partner (Fahs, 2009; Levy, 2005). Described as “performative bisexuality” (Fahs, 2009) or “heteroflexibility” (Diamond, 2005), such behaviors have become part of the script of heterosex, satisfying men’s sexual fantasies of women having sex with each other (Kimmel & Plante, 2002), without challenging the hegemony of compulsory heterosexuality. This is because these same-gender sexual experiences do not preclude women’s self-identification as heterosexual, either at the time, or in retrospect (Diamond, 2003; Lamb, 2004). Women college students describe themselves as “lesbian until graduation” (LUG) and “bisexual until graduation” (BUG) (Plante, 2015), indicating the temporal location of same-gender sexual practices. This temporality is also reflected in popular cultural depictions of women exploring same-gender sex and then reverting to being heterosexual, or not questioning their heterosexual identity at all (Fahs, 2009). It is thus of no surprise to find that heterosexually identified women participating in the research Chivers’ reviews respond to sexual images of other women, or to lesbian sex. They have learned to eroticize such representations and practices, yet still define themselves as heterosexual.

## Deconstructing Sexual and Gender Identities: Destabilizing Heteronormativity

The influence of positivism/realism is also evident in Chivers’ adoption of the reductionist term “sexual orientation,” the use of the Kinsey Scale to measure orientation, and the concepts of “gynephilia,” “ambiphilia,” and “androphilia” to categorize individuals, based on their gendered sexual response. This serves to position sexual identities as internal, stable, and fixed, negating the complexity of sexual desire and response, and the potential fluidity and multiplicity of sexual subjectivity. The implicit rejection of established sexual identity labels, such as heterosexual, bisexual, and lesbian, reduces same-gender sexual experience to bodily response that can be “directly assessed” by researchers, positioned as superior to self-identification, a “less accurate indicator of gendered sexual attractions,” in Chivers’ words.

Sexual orientation or identity is a not a biological phenomenon. It is a social construction, located in specific cultural and historical contexts (Foucault, 1978; Valdes, 1995). The concepts of “homosexuality” and “heterosexuality” were first used in the late 1800s; prior to this time, men and women engaged in same-sex activities without adopting a specific sexual identity label (Plante, 2015; Ussher, 1997). This is still the case in some non-Western cultural contexts today. For example, among the Sambia in New Guinea, there is no concept of “heterosexuality” and “homosexuality,” with men expected to engage in sexual activities with both women and men as part of their normal sexual lives (Herdt, 1997). In contemporary Western society, individuals whose sexual desires and practices challenge the hegemony of compulsory heterosexuality may adopt a range of sexual identity labels, including lesbian, dyke, gay, bisexual, queer, questioning, and gender–queer (Robinson, Bansel, Denson, Ovenden, & Davies, 2014). Others engage in same-gender sex, but identify as heterosexual, as outlined above, demonstrating the potential disconnect between sexual identity and sexual desire or activity (Diamond, 2003). Sexual identity positioning may also change over time, with women’s same-gender sexuality being described in terms of “intimate careers,” rather than fixed traits or desires (Peplau, Spalding, Conley, & Veniegas, 1999). Within social constructionist-influenced queer theory, sexual identities are thus considered to be performative behaviors (Butler, 1990), with analysis of such performativity serving to “denaturalize the sexual subject and sexual subjectivity” (Alexander & Anderlini-D’Onofrio, 2012), through documenting “incoherencies in the allegedly stable relations between chromosomal sex, gender and sexual desire” (Jagose, 1996).

There is evidence that the meaning and experience of sexual identity positioning is different for women and men. Women’s sexual identities have been reported to be more situation dependant and less “category specific” than men’s (Diamond, 2008), with women’s sexual desire and response more strongly shaped by sociocultural factors (Baumeister, 2000), and relationship context having a greater influence on young women’s sexual desire and behavior than on young men’s (Hyde & Durik, 2000; Udry, Talbert, & Morris, 1986). Women demonstrate a greater willingness to engage sexually with other women (Fahs, 2009) and are more likely than men to report that their sexuality is fluid and that same-gender attraction or identity is chosen, rather than biologically given (Diamond, 2003). In contrast to the widespread acceptance of women’s same-gender sexual exploration (Fahs, 2009) and the positioning of lesbians as “cool” (Pascoe, 2007), the specter of homosexuality in “fag talk” is used to establish and police heterosexual masculine identities (Pascoe, 2007). Hegemonic masculinity requires the feminine to be renounced (Connell, 1995), with heterosexual men being more negative about gay and bisexual men than about lesbian and bisexual women (Horn & Nucci, 2003). Popular culture and heterosexual pornography is devoid of explicit homoerotic imagery between men, and heterosexually identified men respond negatively to such representations (Bishop, 2015). It is thus no “mystery” that heterosexual men exhibited a gender-specific sexual response in the research Chivers’ reviews—to respond positively to homoerotic imagery is to threaten their very identity as heterosexual men.

## A Material–Discursive–Intrapsychic Understanding of Women’s Sexual Response

My intention in this commentary is not to dismiss the research conducted by Chivers and her colleagues, or the resulting theoretical explanations she draws upon in her review-this work provides one part of a jigsaw that can help us to understand the complexity of women’s sexuality and sexual response. However, the narrow positivist–realist gaze adopted in this work limits the conclusions that can be drawn and the broader social applicability of this work in understanding sexual subjectivity. Acknowledgement of a social constructionist perspective opens up alternative explanations for the finding of “androphilic” women’s gender-non-specific sexual response. This is not to dismiss the materiality of embodied sexual response, or the intrapsychic variables outlined in Chivers’ review. I would suggest that sex researchers adopt a material–discursive–intrapsychic (MDI) model (Gilbert et al., 2013; Ussher, 2000), within a critical realist epistemology (Bhaskar, 1989), in order to acknowledge the interconnections between the materiality of the sexual body, women’s intrapsychic experience of sex and sexual response, and discursive constructions of sex and sexual identity. Within this perspective, women’s sexual response is not positioned as the product of biology nor is it seen as static. Rather, the material body is positioned as inseparable from women’s interpretations and experiences of sexual response and identity, and emphasis is given to how discourses stemming from medicine, psychology, religion, and popular culture define and normalize women’s sexuality and the parameters of sexual response (Ussher, 1997, 2011). Finally, from a methodological point of view, the utilization of qualitative methods alongside experimental measurement would provide insight into women’s perception and experience of the imagery that elicits a sexual response, as well as the meaning of sex and sexual identity. The “mystery” of heterosexual women’s gender-non-specific response could thus be cleared up by simply talking to them.
